# Travel history and malaria infection risk in a low-transmission setting in Ethiopia: a case control study

**DOI:** 10.1186/1475-2875-12-33

**Published:** 2013-01-24

**Authors:** Joshua O Yukich, Cameron Taylor, Thomas P Eisele, Richard Reithinger, Honelgn Nauhassenay, Yemane Berhane, Joseph Keating

**Affiliations:** 1 Department of Global Health Systems and Development, Tulane University School of Public Health and Tropical Medicine, 1440 Canal St, New Orleans, LA, USA; 2 ICF International, Beltsville Drive, Calverton, MD, USA; 3 United States Agency for International Development, Addis Ababa, Ethiopia; 4 RTI International, Washington, DC, USA; 5 Addis Continental Institute of Public Health, Kirikos sub-City, Addis Ababa, Ethiopia

**Keywords:** Malaria, Travel, Human movement, Importation, *Plasmodium vivax*, *Plasmodium falciparum*, Ethiopia, Reservoir infection

## Abstract

**Background:**

Malaria remains the leading communicable disease in Ethiopia, with around one million clinical cases of malaria reported annually. The country currently has plans for elimination for specific geographic areas of the country. Human movement may lead to the maintenance of reservoirs of infection, complicating attempts to eliminate malaria.

**Methods:**

An unmatched case–control study was conducted with 560 adult patients at a Health Centre in central Ethiopia. Patients who received a malaria test were interviewed regarding their recent travel histories. Bivariate and multivariate analyses were conducted to determine if reported travel outside of the home village within the last month was related to malaria infection status.

**Results:**

After adjusting for several known confounding factors, travel away from the home village in the last 30 days was a statistically significant risk factor for infection with *Plasmodium falciparum* (AOR 1.76; *p*=0.03) but not for infection with *Plasmodium vivax* (AOR 1.17; *p*=0.62). Male sex was strongly associated with any malaria infection (AOR 2.00; *p*=0.001).

**Conclusions:**

Given the importance of identifying reservoir infections, consideration of human movement patterns should factor into decisions regarding elimination and disease prevention, especially when targeted areas are limited to regions within a country.

## Background

Malaria is the leading cause of morbidity in Ethiopia, with more than one million clinical cases of malaria reported annually [1]. Control tools such as indoor residual spraying of households with insecticide (IRS) and insecticide-treated mosquito nets (ITNs) are highly effective at reducing exposure to infectious mosquito bites, and the concomitant burden of malaria disease [2-5]. However, existing individual level control measures (e.g, ITNs) do not always target individuals out late at night, moving at peak biting times, or away from personal control measures (i.e, away from ITN and IRS protection at home). The implication for malaria prevention and control is that mobile sub-groups of the population are at increased risk of infection and may also be reservoirs for sustaining malaria transmission in areas where transmission is very low [6-10].

There is evidence to suggest that human movement, interacting with vector habitat and features of the environment may be important for the epidemiology of malaria [11,12]. People locate in time and space between areas of high risk and low risk both at the macro (e.g, regional or district level) and micro-scales (e.g, community or household level), thus exposing them differentially to parasites, different mosquito biting intensity patterns, and potentially different living or environmental conditions that may require alternative protective measures. Previous research has also shown that a history of travelling was a risk factor for *Plasmodium falciparum* malaria infection in refugee settings, coastal areas, urban areas and river basins [13-16], reinforcing the importance of investigations into how human movement may be interacting with malaria infection incidence. Past evidence from the study area in Ethiopia shows that there may be meaningful heterogeneity between villages located at small geographic distances from each other [17]. This implies that human movement, even at small spatial scales, could act as a risk factor for exposure to malaria infection. Differences in risk of malaria infection within the study area may be driven by heterogeneity in human factors, climate factors, altitude or the vector factors as well as by the built environment including urbanization and irrigation schemes [17,18].

As malaria transmission continues to fall in many parts of Africa due to the continued scale-up of malaria prevention and control efforts, transmission is likely to become increasingly heterogeneous, disproportionately affecting sub-populations in high-risk communities [19]. This could potentially produce reservoirs of infection; these carriers could also potentially move, resulting in the spread of parasite reservoirs into areas where local elimination has already occurred, or thwarting or delaying elimination in other areas. As such, it is important to identify risk factors for infection where transmission is low.

This study explores the role of travel history as a risk factor for malaria parasite infection. Investigating aspects of human movement is applicable to Ethiopia and other settings targeting elimination, as it focuses on identifying factors associated with being a source case of infection.

## Methods

### Study design

**Figure 1:**
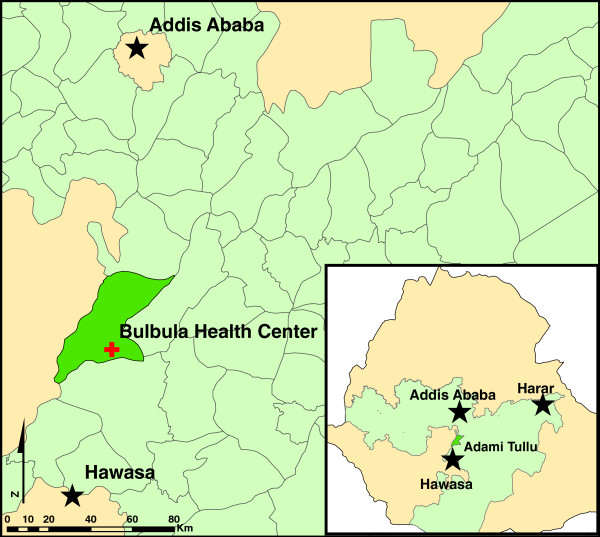
Location of the study site – Bulbulla Health Centre, Adami Tulu Wereda, Oromia Regional State, Ethiopia.

A health facility-based, unmatched case–control study was conducted from 22 June, 2011 to 14 October, 2011. Cases were persons presenting at the Bulbula health facility (Figure 1) with a positive thick blood smear laboratory test or a multi-species rapid diagnostic test (RDT) (CareStart®, Access Bio, NJ, USA), confirming *Plasmodium* infection, regardless of parasite species. Controls were persons presenting at the same facility whose thick blood smear or RDT tests results were negative for infection.

### Study site

Bulbula health centre is a malaria epidemic detection and sentinel surveillance site in Oromia, part of an on-going collaboration between the U.S. Presidents Malaria Initiative, the Ethiopia Health and Nutrition Research Institute, and the Oromia Regional Health Bureau. The health centre is located in Adami Tulu Jido Kombolcha *wereda* (district) (an area of approximately 2,300 km^2^) where altitude ranges from 1,500-2,300 m above sea-level. The health centre is located in a market town with a current population of ~8,500 and provides primary health care services to outpatients and serves as the central point of the primary health care system in the area [20]. The health centre itself is located at an altitude of approximately 1,600 m above sea-level 133 km south of Adama and 182 km south-south-west of Addis Ababa. Travel to major cities is possible using well paved roads, however, the road network between nearby areas and surrounding villages consists mostly of unpaved road or footpath, as such rainy season travel may be significantly more difficult in the area when compared to dry season travel. Most of the surrounding population is engaged in agricultural activities, including the raising of domestic livestock and the growing of *teff*, maize, and vegetables. Agricultural opportunities have also attracted significant numbers of migrant workers to the area [18]. Surveillance data collected during the three years preceding this study indicate that transmission in the Bulbulla area is bi-modal with peaks occurring in June or July and September or October. This data also shows a relatively even mix of *P*. *falciparum* and *Plasmodium vivax* infections (Ethiopia Epidemic Detection and Sentinel Surveillance Project, unpublished data). *Anopheles arabiensis* is the main malaria vector in the country [21,22].

### Inclusion and exclusion criteria

Inclusion criteria for the study were: attendance at the Bulbula Health Centre Out Patient Department (OPD), age 18 years or older, residence in Adami Tullu Jido Kombolcha *wereda*, fever or history of fever in the previous 72 hours, and received a Giemsa stained thick blood smear or RDT for malaria diagnosis at the health centre on the day of recruitment.

Participants were excluded from the study if they were under 18 years of age, extremely ill and in need of immediate medical attention at the time of testing, had self-reported malaria in the previous 30 days (excluding the current episode), had taken malaria prophylaxis or treatment in the previous 30 days, or were not full-time residents of Adami Tullu Jido Kombolcha *wereda*.

### Sample size determination and recruitment

Sample size calculations were conducted in the StatCalc module of EpiInfo (EpiInfo™ version 3.5.1, U.S. Centers for Disease Control and Prevention, Atlanta, GA, USA). The study was powered to detect an odds ratio of 2.0 with 95% significance and 80% power on the measure of having travelled and spent a night away from the home (outside the immediate community) in the previous 30 days, assuming that 20% of those who were not infected with any parasite species had travelled overnight in the past month, and a 1:4 case–control mix. In total, 560 patients were recruited for the study. These consisted of 141 cases (positive thick film or RDT for any malaria parasite species) and 419 controls (negative thick film for all malaria species).

### Data collection

Participants were recruited over a 16-week period. After obtaining informed consent, patients were interviewed using a standard, pre-tested questionnaire covering socio-demographic and household risk factors for malaria including recent travel history. Interviews were conducted in either Oromiffa or Amharic, depending on the language preference of the patient. To minimize recall bias, an Ethiopian calendar was supplied for use as a reference for recalling travel dates. When recording the names of the villages where patients travelled, the data collector used a list of kebeles with corresponding village names provided by the Ethiopian Central Statistics Agency and a map of the surrounding area. Patients were first asked about any overnight stays away from their home village in the past 30 days, then respectively asked to identify when and where they had travelled and from which dates with the aforementioned aides. After the interview was complete, the patients were taken back to the laboratory to obtain their malaria test results; participants were then classified as either a case or a control. Data were double entered in Microsoft Access (Microsoft Corporation, Redmond, WA, USA) database and converted to STATA 10.0 (Stata Corporation, College Station, TX, USA) format for analysis.

### Data analysis

Chi-square (χ^2^), Fischer’s exact tests, and multivariate logistic regression analysis was conducted to test whether travel was a risk factor for malaria. Multivariate logistic regression analysis was used to control for age, sex, socio-economic status and use of protection methods such as bed nets. Sub-analyses of cases identified as *P*. *falciparum* infection *vs* no *P*. *falciparum* infection, and *P*. *vivax* infection *vs* no *P*. *vivax* infection were conducted. Mixed infections (n=1) were included as cases in both sub-analyses. A simple wealth index was created using binary variables for durable asset possession using principal components analysis (PCA) [23]. Only the first principal component was included [24]; the patient population was then categorized into wealth quartiles.

### Ethical approval

Institutional Review Board (IRB) approval was obtained from Tulane University in New Orleans, Louisiana, USA and the IRB of the Addis Continental Institute of Public Health in Addis Ababa, Ethiopia.

## Results

### Description of study sample

A total of 560 patients were successfully recruited for the study: 141 cases and 419 controls. Of the 141 infected individuals, 85 (60.3%) and 57 (40.4%) were diagnosed with *P*. *falciparum* or *P*. *vivax* malaria, respectively; only one patient had a mixed infection.

The sample contained more females than males (54.7% *vs* 44.7%) (Table 1). The age of study participants ranged from 18–80 years of age with the majority of the patients in the study being between the ages of 18–25 years. The most frequent occupation among study participants was agricultural work.

**Table 1:** Basic description of the study sample

	***P*.** ***f*** **+**^**§**^	***P*.** ***v*** **+**^**§**^	**All positive**	**Negative**	**Total**
	**n=85 (%)**	**n= 57 (%)**	**n=141 (%)**	**n= 419 (%)**	**n=560 (%)**
**Sex**					
Female	39 (45.9)	22 (38.6)^**^	60 (42.6)^**^	247 (59.4)	307 (54.8)
Missing	0 (0.0)	0 (0.0)^**^	0 (0.0)^**^	3 (0.7)	3 (0.5)
**Age Category (years)**					
18-20	26 (30.6)	24 (42.1)	50 (35.5)	116 (27.7)	166 (29.6)
21-25	17 (20.0)	12 (21.1)	29 (20.6)	80 (19.1)	109 (19.5)
26-32	16 (18.8)	11 (19.3)	27 (19.2)	113 (27.0)	140 (25.0)
33+	23 (27.1)	9 (15.8)	31 (22.0)	100 (23.9)	131 (23.4)
Missing	3 (3.5)	1 (1.8)	4 (2.84)	10 (2.4)	14 (2.5)
**Occupation**					
Agriculture	62 (72.9)	38 (66.7)	99 (70.2)	301 (71.8)	400 (71.4)
Non-Agricultural	23 (27.1)	19 (33.3)	42 (29.8)	116 (27.7)	158 (28.2)
Missing	0 (0.0)	0 (0.0)	0 (0.0)	2 (0.5)	2 (0.4)
**Own Animals**					
Yes	76 (89.4)	47 (82.5)	122 (86.5)	365 (87.1)	487 (87.0)
**Roof Material**					
Thatch/Leaf	53 (62.4)^*^	27 (47.4)	79 (56.0)	206 (49.2)	285 (50.9)
Corrugated Iron	31 (36.5)^*^	30 (52.6)	61 (43.3)	205 (48.9)	266 (47.5)
Other	1 (1.2)^*^	0 (0.0)	1 (0.7)	8 (1.9)	9 (1.6)
**Wall Material**					
Bamboo/Wood	28 (32.9)	19 (33.3)^**^	47 (33.3)^*^	140 (33.4)	187 (33.4)
Covered Adobe	38 (44.7)	15 (26.3)^**^	53 (37.6)^*^	200 (47.7)	253 (45.2)
Wood Planks/Shingles	12 (14.1)	13 (22.8)^**^	24 (17.0)^*^	44 (10.5)	68 (12.1)
Other	7 (8.2)	10 (17.5)^**^	17 (12.0)^*^	33 (7.9)	50 (8.9)
Missing	0 (0.0)	0 (0.0)^**^	0 (0.0)^*^	2 (0.5)	2 (0.4)
**Electricity**					
Yes	15 (17.7)	17 (29.9)	32 (22.7)	95 (22.7)	127 (22.7)
Missing	1 (1.2)	0 (0.0)	1 (0.7)	3 (0.7)	4 (0.7)
**Radio**					
Yes	29 (34.1)	21 (36.8)	50 (35.5)^*^	190 (45.4)	240 (42.9)
Missing	1 (1.2)	1 (1.8)	2 (1.4)^*^	4 (1.0)	6 (1.1)
**Travel Overnight**					
Yes	36 (42.4)	21 (36.8)	57 (40.4)	136 (32.5)	193 (34.5)
Missing	0 (0.0)	1 (1.8)	1 (0.7)	6 (1.4)	7 (1.3)
**Owned an ITN**					
Yes	34 (40.0)	19 (33.3)	53 (37.6)	181 (43.2)	234 (41.8)
Missing	1 (1.2)	0 (0.0)	1 (0.7)	6 (1.4)	7 (1.3)
**Slept under ITN previous night**					
Yes	14 (16.5)	12 (21.1)	26 (18.4)	82 (19.6)	108 (19.3)
Missing	1 (1.2)	1 (1.8)	2 (1.4)	9 (2.2)	11 (2.0)

^§^*P*.*f*.+ and *P*.*v*.+ totals include mixed diagnosis; ^**^Significant difference between cases and controls at the 5% level, using a χ^2^ (Fischer’s Exact) test. ^*^Significant difference between cases and controls at the 10% level using a χ^2^ (Fischer’s Exact) test.

Patients self-reported that their homes were primarily roofed with thatch/leaf or corrugated iron, while wall structures were largely reported to be covered with adobe or wood. Adami Tulu Jido Kombolcha is a largely rural area, where most people are engaged in farming and other agricultural activities as a means of subsistence, as such, 87% of study participants report owning animals, largely cattle, donkeys or sheep.

### Univariate analysis

#### Travel history

One hundred ninety four study subjects (35%) reported staying overnight outside of their home village for at least one night during the previous 30 days. A total of 203 overnight trips were reported by these persons. Nearly all travellers made only one trip overnight during the previous 30 days. Among those reporting an overnight stay, the median length of stay was three days (Interquartile range 1–28 (mean 5.2 days)). Travel was more common among men than among women (41% *vs* 30%; Odds Ratio (OR)=1.65, *p*=0.005). Most trips (67%) were made to areas within Adami Tulu *wereda*. Travel history was associated with malaria infection, but the relationship was only of borderline significance, regardless of infecting parasite species.

#### Bed net ownership and use

Two hundred and thirty four (41.8%) study participants reported that their household had at least one ITN. Of those who reported owning an ITN, 108 (46.2%) reported having slept under an ITN the night before the interview, meaning that overall, only 19.3% of all study participants had used a net the night before they enrolled in the study. Only 5% of patients with a history of travel reported ITN use during their travel. Sleeping under an ITN did not appear protective against malaria in this population and was not associated significantly with being either a case or control (44% *vs* 38%; *p*=0.217).

#### Malaria infection

The vast majority (519 or 93%) of laboratory confirmed malaria infections were confirmed with microscopy, the remainder of laboratory confirmations (41 or 7%) used RDTs. No significant differences were found between the odds of infection using either method. Men were more likely to be diagnosed with malaria of all types than women (OR=1.97, 95% CI: 1.34–2.91), and *P*. *vivax* in particular (OR=2.11, 95% CI:1.20–3.70). Patients who lived in houses with corrugated iron roofs were less likely to be infected with *P*. *falciparum* than those living in houses with other roof types (OR=0.59, 95% CI:0.36–0.94). No statistically significant relationship of roof type with *P*. *vivax* infection was seen (OR=1.25, 95% CI: 0.73–2.18). Having a wall type other than covered adobe appeared to be a strong predictor of *P*. *vivax* (OR= 2.53, 95% CI: 1.37–4.69), but not for *P*. *falciparum* infection.

#### Multivariate analyses

Logistic regression models were fit with *P*. *falciparum*, *P*. *vivax* and all malaria cases as outcome variables to adjust for potential confounding. Known and measured potential confounders included sex, age, socio-economic status, household characteristics, and use of preventative measures (ITNs); all variables were included in the final logistic regression models. The results of the analysis are shown in Table 2. Individuals with a *P*. *falciparum* diagnosis were more likely to have travelled in the previous 30 days than controls (Adjusted Odds Ratio (AOR)=1.76, 95% CI: 1.06–2.93). This was also true for all malaria cases (AOR=1.64, 95% CI: 1.07–2.52), but not for *P*. *vivax* infections alone (AOR 1.17, 95% CI: 0.63–2.16). Age appeared to be protective for *P*. *vivax*, but not for *P*. *falciparum* infection, while wealth was associated with reduced odds of *P*. *falciparum* infections alone. Male sex, even after adjusting for travel, household factors, SES and use of a protective measure, was associated with *P*. *vivax* infection (AOR=2.01, 95% CI: 1.09–3.71).

**Table 2:** Adjusted odds ratios from logistic regression models

	***P*.** ***f*.+**		***P*.** ***v*.+**		**All cases**	
**Characteristic**	**AOR (95% CI)**	***p*-value**	**AOR (95% CI)**	***p*-value**	**AOR (95% CI)**	***p*-value**
**Travel**	1.763^**^	0.030	1.17	0.618	1.64^**^	0.024
	(1.06-2.93)		(0.63-2.16)		(1.07-2.52)	
**Wealth (Quartile)**						
*Most Poor* (1)	-	-	-	-	-	-
(2)	0.72	0.329	1.47	0.386	0.97	0.904
	(0.38-1.39)		(0.61-3.52)		(0.54-1.71)	
(3)	0.46^**^	0.029	1.09	0.846	0.60^*^	0.098
	(0.23-0.92)		(0.45-2.66)		(0.33-1.10)	
*Least Poor* (4)	0.36^**^	0.008	1.26	0.609	0.57^*^	0.067
	(0.17-0.76)		(0.52-3.02)		(0.31-1.03)	
**Age Category (years)**						
18-20	-	-	-	-	-	-
21-25	1.12	0.751	0.66	0.289	0.85	0.581
	(0.56-2.24)		(0.30-1.43)		(0.48-1.51)	
26-32	0.69	0.299	0.45^**^	0.050	0.51^**^	0.021
	(0.34-1.39)		(0.21-1.00)		(0.29-0.90)	
32+	0.94	0.854	0.27^**^	0.006	0.49^**^	0.018
	(0.48-1.84)		(0.10-0.68)		(0.27-0.89)	
**Sex**						
Female	-	-	-	-	-	-
Male	1.62^*^	0.065	2.01^**^	0.025	2.00^**^	0.001
	(0.97-2.70)		(1.09-3.71)		(1.31-3.06)	
**Slept under ITN the night prior survey**	0.89	0.732	1.32	0.455	1.09	0.752
	(0.45-1.75)		(0.64-2.75)		(0.64-1.87)	

^§^*P*.*f*.+ and *P*.*v*.+ totals include mixed diagnosis; ^**^Significant difference between cases and controls at the 5% level. ^*^Significant difference between cases and controls at the 10% level.

## Discussion

While travel to a malaria-endemic area in Ethiopia is recognized as a risk factor for malaria infection, much of the country is endemic for malaria transmission and little attention has been paid to whether or not routine human movement patterns can lead to higher risks of malaria infection, even on a small geographic scale within endemic areas. This study shows that travel may be a risk factor for symptomatic *P*. *falciparum* infection even when the travel occurs within endemic areas. Such endemic areas, including the area where this study was conducted, may vary in risk of infection even within relatively small geographic zones, for reasons which may related to environmental factors as well as a myriad of other influences [17,18]. Thus the relationship of travel to malaria risk is likely due to a combination of factors including movement into areas of higher transmission during risk periods for mos-quito biting, such as movement for harvesting of crops which correlates with the transmission season in Ethiopia, relaxed use of preventative measures when individuals are away from home, and possible behavioural differences by sex or during travel. Ethiopia is home to a large pastoralist population which frequently moves in search of pasturage for animals, and further other short-term travel to visit nearby market areas, friends and relatives or for school attendance is also common. No distinction between travel to areas with differing malaria risk was made in this analysis, a fact which could lead to bias in the estimate of travel history as a risk factor compared with assessing travel history to a higher malaria risk area. Such a bias would be expected to be in the direction of the null hypothesis because including areas with low or no malaria risk in the analysis should reduce the overall association of malaria infection and travel history.

In this study blood slides and RDTs were performed only once, as such misclassification bias could arise due to the potential for inaccurate microscopy readings by facility technicians. However, the facility was part of an on-going quality assurance and control programme (QA/QC) at the time of the study. The contemporaneous QA/QC results showed 96% agreement of facility readings with expert readings over the five month period surrounding the study with regards to positivity and negativity, and 90% agreement on species identification. This suggests that any misclassification bias associated with microscopy conducted at the facility level is likely to be small, furthermore, such a bias would likely be in the direction of the null hypothesis.

Within low transmission areas, importation of infections could lead to maintenance of reservoirs of infection in the absence of local vectorial capacity to maintain transmission [25,26]. Understanding where and how infections originate in the Ethiopian context will be important for assessing prospects for local transmission interruption. The data in this study show that it may be important to consider importation as a potential source of *P*. *falciparum* infections, even within moderately endemic areas of the country (Bulbulla health centre’s estimated prevalence (*Pf*PR_2-10_ based on the Malaria Atlas Project is approximately 7%) [27].

As *P*. *vivax* possesses a dormant hypnozoite stage and causes relapses of malaria, many of the *P*. *vivax* infections in this study may have originated not from initial sporozoite inoculations, but rather as relapses. The epidemiology of the disease appears different among *P*. *vivax* infections in this study, travel, at least recent travel, was not a marked risk factor, while age and sex were important. In this study male sex was the greatest risk factor for all categories of malaria infection. It is possible that behavioural factors associated with being male (e.g, staying out late in the evening) could be predisposing towards malaria infection. While men in this study were more likely to travel than women, and spend the night away from their home village, even after adjusting for this travel, sex remained a significant risk factor for all types of malaria infection.

In this study, ownership or use of an ITN showed no statistically significant protective effect against *P*. *falciparum*, and was associated with increased infection with *P*. *vivax*. While this finding is out of line with the literature and large-scale evaluation research, lack of protection from net use has been reported in observational studies in Ethiopia before [28-31]. In this context, these results could derive from a combination of factors including the prevalence of old and ineffective nets, the epidemiology of *P*. *vivax* infection, endogeneity between the use of nets and malaria risk or simply the limited sample size.

In order to assess effect-modification through the relaxed use of protective measures during travel the interaction between net use and travel was tested. Though the interaction term was statistically insignificant in this context, this was possibly hampered by small sample size. Regardless, the low level of use of LLIN use indicates the need to focus on developing a strong distribution system to maintain LLIN coverage over time, and to increase use among those travelling and staying away from their home overnight.

While this study indicates travel is a risk factor for *P*. *falciparum* infection, the study is a small case–control study of limited geographic scope. Because this study focused only on patients presenting at one health centre, the study has limited external validity. Data collection from national parasitemia surveys or at a minimum from multiple health centres would help to make these results more generalizable. Further, the size of the adjusted odds ratios found indicate that travel is not a strong risk factor. However, given that the study area has higher transmission than some other areas of Ethiopia, notably the Rift Valley basin near Eritrea or highland areas, travel may be an even more important factor in other areas of the country [27]. Travel history is also a potentially difficult variable to measure retrospectively; patients may not remember the dates or duration of their travel accurately over long recall periods. For this reason questions on travel were limited to the 30 days before the administration of the questionnaire. However, recall bias may still be present. Further, this study did not attempted to differentiate between travel in the previous week and travel occurring more than a week previously, which might help to focus results on the period during which an infection would have to have occurred in order to produce current symptoms.

The epidemiology of *P*. *vivax* is generally poorly explored [32] and given the current lack of a sound method for distinguishing relapses from current infections this study cannot distinguish between travel as a risk factor for new infection, likely diluting the potential effects. Nevertheless, *P*. *vivax* transmission is likely to be more resilient to interventions that reduce transmission pressure for several reasons including greater and more rapid production of gametocytes, shorter extrinsic incubation periods and the dormant liver stage [32]. As such travel might be expected to be less of a factor in the acquisition of new *P*. *vivax* infections in this setting but could easily contribute to the maintenance of transmission even in very low transmission areas.

Derivation of travel history from individual recall is likely to be subject to recall bias. Recent technological developments including inexpensive small Global Positioning System data loggers and mobile phones may give rise to the ability to correct for these biases to some extent, by allowing for relatively fine scale temporal and spatial tracking of human or at least device movement [33,34]. The results of this study indicate that increased risk of infection may arise not only from the movement of individuals themselves but also from changes in behaviour coupled with travel. Such behaviour changes cannot currently be tracked using mobile phone databases or GPS logging systems and as such individual survey methods may still be required to fully understand the epidemiology of malaria infection and its interaction with travel and human movement patterns.

These findings suggest that careful consideration should be given to travellers and perhaps especially adult men in areas of moderate transmission in Ethiopia. These individuals appear to be at the highest risk of malaria infection. While this study cannot determine if the risk evolves from travel to areas of higher risk or relaxation of behavioural preventative measures during travel, it indicates the potential for interventions on communicating the importance of maintaining behavioural prevention activities, such as use of bed nets during travel. It additionally suggests that such communication measures might be most advantageous if targeted towards men.

## Conclusions

Travel is a risk factor for *P*. *falciparum* infection in a highland fringe area of Oromia, Ethiopia while male sex is a risk factor for all types of malaria infection. Men travel more frequently overnight than women, importation of infections may be an important contributor to maintenance of transmission even in relatively high transmission areas for Ethiopia. Further investigation quantifying the role of importation of infections to endemic areas could contribute to programmatic strategies to locally interrupt transmission in Ethiopia.

## Competing interests

The authors declare that they have no competing interests.

## Authors’ contributions

JY, JK and CT conceived the study, developed the methods, conducted the analysis and drafted the manuscript. TE and RR contributed to study development and drafting of the manuscript. HN, YB and CT contributed to the development of study methods, collected the data, and critically read and contributed to the drafting of the manuscript. All authors read and approved the final manuscript.
